# Development and validation of educational multimedia to promote public health literacy about healthy cognitive aging

**DOI:** 10.1111/hex.13857

**Published:** 2023-08-27

**Authors:** Aysha Rooha, Shreya Shetty, Gagan Bajaj, Nidhi L. Jacob, Vinitha M. George, Jayashree S. Bhat

**Affiliations:** ^1^ Department of Audiology and Speech Language Pathology Kasturba Medical College, Mangalore, Manipal Academy of Higher Education Manipal Karnataka India; ^2^ Department of Audiology and Speech Language Pathology National Institute of Speech and Hearing Trivandrum Kerala India; ^3^ Department of Audiology and Speech Language Pathology Nitte Institute of Speech and Hearing, Deralakatte Mangalore Karnataka India

**Keywords:** cognition, health education, health promotion, multimedia, patient education

## Abstract

**Objectives:**

Health literacy (HL) about healthy cognitive aging is essential in preventing cognitive decline and promoting cognitive well‐being. It is important that one such HL module should be scientifically designed, delivered in a technically sound manner to the audience, and specific to the context. The present study thus aimed at developing and validating educational multimedia about cognitive health.

**Methods:**

The study followed a methodological framework and was carried out across three phases, that is, identification of themes, development and validation of educational script and design, and validation of educational multimedia. The module was scripted based on the Integrated HL framework and the recommendations made during the modified nominal group technique among the research team. Seven speech‐language pathologists (SLPs), with expertise in the field of cognitive sciences, and 15 representatives of the general public validated the module using the Educational Content Validation Instrument in Health and the Patient Education Materials Assessment Tool for Audiovisual Materials questionnaire.

**Results:**

The scientific content of the educational script received satisfactory agreements among the experts (content validity index [CVI]: 0.93) and representatives of the general public (CVI: 0.86). The technical aspects of the educational multimedia were rated to have high understandability (experts: 92.8%; representatives of general public: 98.8%) and actionability (experts and representatives of general public 100%).

**Conclusion:**

Overall, the developed educational multimedia scored optimally with respect to the objective, structure, relevance of the content, actionability and understandability of the multimedia. The developed module holds the potential to be used at community and national level health educational programs or awareness campaigns to enhance public knowledge and beliefs pertaining to cognitive health.

**Patient or Public Contribution:**

SLPs with expertise in the field of cognitive science and representatives from the general public were included to validate and obtain feedback on the developed educational multimedia.

## 1 INTRODUCTION

Health literacy (HL) is a key indicator of health outcomes. It refers to people's knowledge, interest, comprehension, evaluation and use of health information when making daily assessments and decisions regarding healthcare, prevention of disease and health improvement to preserve or enhance their quality of life.^1^ HL is becoming more crucial as the healthcare system becomes more complex, especially in an aging population.^2^ Low HL carries a high risk of health inequalities among aging individuals, poorer health outcomes, chronic disease, lower participation in preventive care, increased healthcare utilization, mortality and reduced quality of life.^3^, ^4^, ^5^, ^6^ As such, HL has been considered a global public health objective in the 21st century to improve health outcomes and promote health promotion through improved education and communication strategies.^7^ In this regard, researchers have developed HL programs to advance awareness of health conditions in various areas of healthcare like cancer, diabetes, physical health, and smoking habits.^8^, ^9^, ^10^ These studies have advocated for the substantial role of HL programs in improving health‐related knowledge and beliefs. Another relevant healthcare domain where the role of HL is warranted and is being researched is cognitive health.

Cognitive health is becoming increasingly important, given the exponential rise in the current and predicted prevalence of cognitive deficits like dementia.^11^ As per the GBD 2019 Dementia forecasting collaborators, 57.4 million people have dementia worldwide as of 2019, with the number expected to rise to 152.8 million by 2050.^12^ According to the ‘Dementia in India—2020’ research, 14 million Indians over 60 were predicted to have dementia by 2050, up from the current estimate of 5.3 million.^13^ Dementia has been recognized as a public health priority by most national and international organizations (World Health Organization, Alzheimer's Disease International, Global Alzheimer's and Dementia Action Alliance). Thus, increasing public awareness regarding cognitive health has been emphasized as one of the most important goals of the ‘Global Action Plan on the public health response to Dementia’.^14^ In this regard, studies have shown a direct and reciprocal link between HL levels and cognitive functions.^5^ Poor cognitive performance has been associated with low HL and may eventually be the cause of it. Individuals with low HL have also been reported to experience a noticeably faster loss of cognitive ability over time.^15^, ^16^ Conversely, those with higher HL and education levels tend to engage more in cognitive, social and physical activities,^17^, ^18^, ^19^ which may indirectly assist in preventing age‐linked cognitive decline. Increasing knowledge about cognitive health may have a positive influence on one's ‘need for cognition’, that is, the desire to engage in cognitively demanding activities.^18^ Further, adequate knowledge may also aid in the early detection of cognitive deviations.^3^ Thus, cognitive HL could play a significant role in promoting cognitive wellbeing.

A recent systematic review revealed that dementia literacy interventions improve self‐efficacy among nonhealth professionals in managing and caring for individuals with dementia and their knowledge of dementia.^20^ However, the review concluded that more evidence is needed linking to dementia beliefs, attitudes and preventive behaviour. Five‐week intervention program in health education by Du and Hu^21^ was reported to be successful in enhancing the knowledge about Alzheimer's disease (AD) and encouraging healthy lifestyle among Chinese older adults without AD. Another study examining the efficacy of Preventive Dementia Massive Open Online Course among global participants suggested that the program improved self‐efficacy and understanding of dementia risk reduction, higher willingness to maintain healthy behaviours, and improved use of the knowledge to modify health behaviour change which could potentially lower the risk of developing cognitive disorders.^22^ These studies reinforce the successful role of HL programs in influencing cognitive health‐related knowledge and beliefs. The majority of existing studies focus on dementia literacy, that is, risk factors for dementia, signs and symptoms of dementia, and assessing and managing dementia. HL programs focusing on healthy cognitive aging may help promote a healthy cognitive lifestyle.

According to the Centers for Disease Control and Prevention, consumers have little access to information regarding cognitive health, and perceptions of brain health vary by ethnicity, culture and geography.^23^ Language and culture give the experience background for understanding health information.^24^ The way that a person receives healthcare information is influenced by culturally ingrained ideas, values and preferences that they have. In the Indian context, a recent survey conducted on the general public and healthcare personnel revealed that adults had vague to average knowledge about dementia with poor attitudes and practices towards dementia preventive strategies.^25^ However, there exists a dearth of scientifically developed educational HL modules for enhancing cognitive health among the Indian population. This, along with alarming dementia statistical predictions, warrants the need to develop cognitive HL programs within the Indian context. Thus, the present study aimed at developing and validating an educational multimedia program about cognitive health.

A multimedia mode was chosen for the present study as it has been proven to be more effective in capturing participants' attention, enhancing their experience, contentment, knowledge and in altering their behaviour with longer retention of information.^26^ Multimedia programs integrate textual, audio and visual elements that support and enrich each other to enhance learning and captivate audiences.^27^ By reducing the need for reading and providing information through multiple modalities, multimedia education programs offer the ability to improve communication and education to those with low HL.^27^

## 2 METHODS

### 2.1 Study design

The present study followed a methodological framework according to the recommendations made by similar studies in other health domains.^28^, ^29^ The study was conducted between January 2022 and February 2023. Ethical approval was obtained from the Institutional Ethics Committee (IEC KMC MLR 05‐2022/171).

### 2.2 Participants

Development of the educational multimedia on cognitive health required a systematic process that involved stakeholders like experts and representatives from the general public. Seven speech‐language pathologists (SLPs) with greater than 5 years of experience in the field of cognitive science were included as experts in this study. Representatives of the general public included five young adults (18–40 years), five middle‐aged adults (41–65 years), and five older adults (66+ years) from middle socioeconomic status (ascertained using modified Kuppuswamy scale^30^), with optimum proficiency in the English language (Minimum score of ‘Seven’ on Language Experience and Proficiency Questionnaire^31^). To minimize any bias in perceptions, it was also ensured that all the general public representatives had no prior association with individuals having Alzheimer's or dementia in their immediate family. The demographics of the participants are provided in Table 1. Informed consent was obtained from all participants at the beginning of the study.

**Table 1:** Participant characteristics.

Characteristic	Experts (*N* = 7)	Representatives of general public (*N* = 15)		
	SLP	YA (*n* = 5)	MAA (*n* = 5)	OA (*n* = 5)
Mean age	34.6 ± 3.91 years	23.2 ± 5.59 years	49.6 ± 5.41 years	71.8 ± 4.21 years
Gender	Male: *n* = 3	Male: *n* = 1	Male: *n* = 1	Male: *n* = 3
	Female: *n* = 4	Female: *n* = 4	Female: *n* = 4	Female: *n* = 2
Education	PhD: *n* = 4	Pursuing graduation: *n* = 3	Bachelor's degree: *n* = 3	Bachelor's degree: *n* = 4
	Master's degree: *n* = 3	Bachelor's degree: *n* = 2	Master's degree: *n* = 2	High school graduate: *n* = 1
Employment	>5 years' experience: *n* = 7	Employed: *n* = 2	Employed: *n* = 4	Retired: *n* = 4

Abbreviations: MAA, middle‐aged adults; OA, old adults; SLP, speech language pathologist; YA, young adults.

### 2.3 Procedure

**Figure 1:**
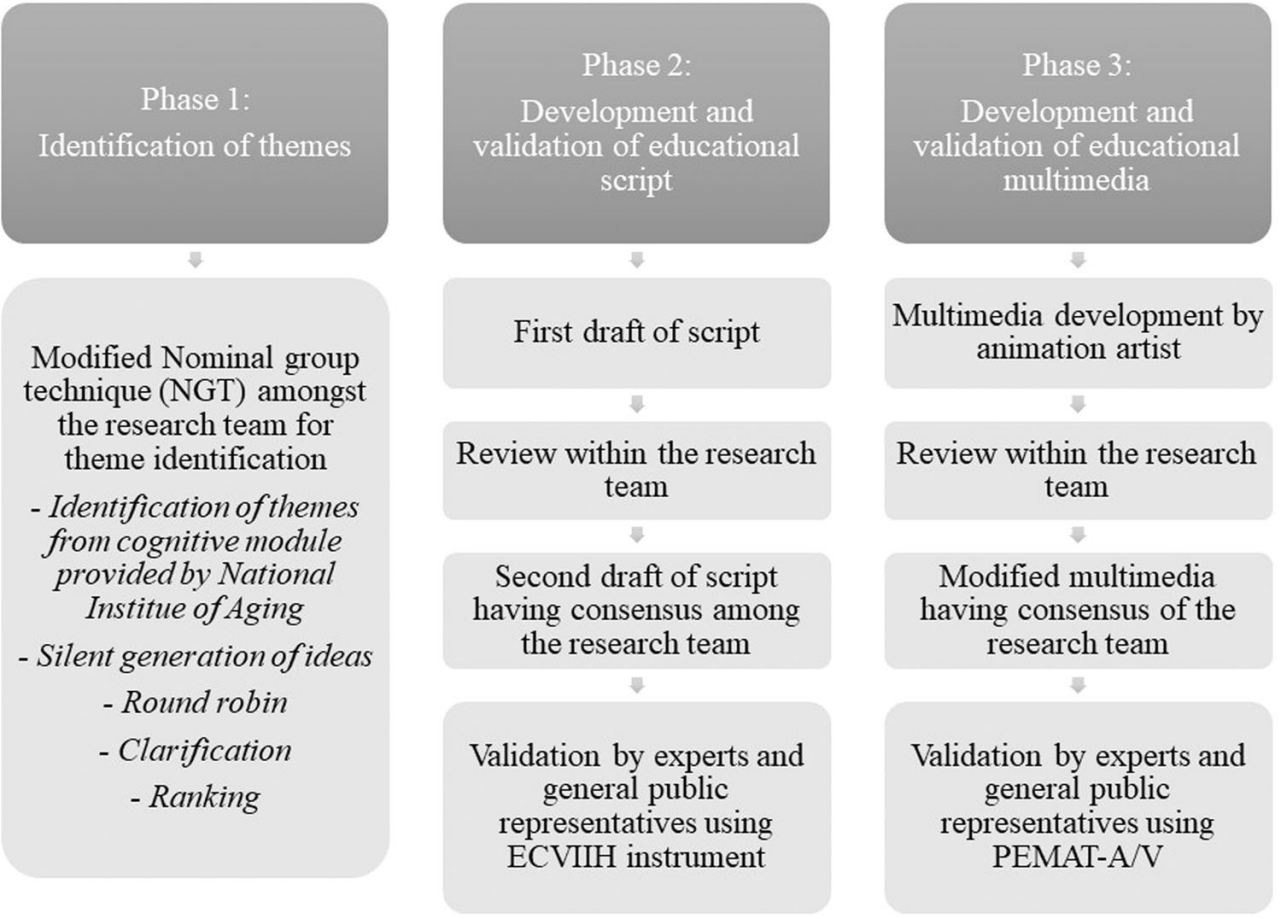
The development and validation phases of the educational multimedia. ECVIIH, Educational Content Validation Instrument in Health; PEMAT‐A/V, Patient Education Materials Assessment Tool for Audiovisual Materials.

The study was conducted in three phases, that is, theme identification, script development and validation, followed by video development and validation (Figure 1).

### 2.4 Phase 1: Identification of themes

The first phase focused on identifying the essential themes for educational multimedia. The integrated HL framework^1^ was used to develop the educational material, as it informs important aspects to consider for users to experience the continuum. A modified nominal group technique (mNGT) was employed to brainstorm and establish consensus on the themes.^32^ The mNGT follows a systematic process wherein the ideas searched from existing literature are combined with the self‐generated ideas of each panel member, followed by clarifications, discussions and voting among them to shortlist the most relevant themes.^32^ The mNGT panel included the authors of the current study, with Author 1 serving as the facilitator and Authors 2–4 as members. Each panelist possessed over 3 years of experience in the field of cognitive science. In the present research, the mNGT was carried out in five steps: literature search and web search for fact sheets on healthy cognitive aging, silent generation of ideas, round robin, clarification and ranking the order of presentation of themes. At the outset, the panelists were explained that the primary objective of this mNGT was to identify the key themes they thought should be addressed in an educational video about healthy cognitive aging for the general public. In the first step, all the important themes in the cognitive health module provided by the National Institute of Aging^33^ were documented. In the following step, silent generation of ideas, panel members were shown all the themes extracted from the literature on a whiteboard and were asked to silently generate additional ideas for themes beyond what was extracted in the first step. Panel members were given up to 20 min to individually reflect on and record their ideas. In the round‐robin step that followed, each member presented their ideas, which were updated on the whiteboard by the facilitator for all to see. No discussions took place during this stage. In the subsequent step, clarification, a group discussion was held to address each idea. Panel members had the opportunity to exclude, include, modify or group similar ideas, reaching an agreement among all members. Consequently, the list of themes was further narrowed. In the final step, known as ranking, each member individually assigned a rank to the themes, determining the order of their presentation in the video. Consequently, the sequence in which the themes had to be scripted was established by considering the theme that obtained the highest rank among all themes. The themes obtained at each step are presented in the results section.

### 2.5 Phase 2: Development and validation of educational script

The second phase focused on drafting the script based on the determined themes following the order established through the ranking process. The first version of the script was written by Authors 1 and 2 in a ‘TED‐like’ style,^34^ which included the main character (Dr. Cognition) giving a talk on cognitive health titled ‘Towards healthy cognitive aging’ to a large audience. The first draft of the script underwent review by the research team (Authors 3–6) during a group discussion. Based on the suggestions of the research team, revisions were made to the script. The revised version was written in a dyadic manner such that it reflected a two‐way interaction between the main speaker, Dr. Cognition, and the audience. The second draft was reviewed again by the research team (Authors 3–6) during another group discussion and a consensus was established among them. The script was then content validated by seven SLPs and 15 representatives from the general public using the Educational Content Validation Instrument in Health (ECVIH).^35^ ECVIH is a widely used tool.^36^, ^37^, ^38^ The instrument has been used to validate educational content presented in various forms, such as multimedia content, print content, digital content and many more. It consists of 18 questions with a focus on the video's objective (intention and aim), structure and presentation (organization), and relevance (significance, impact, motivation and interest). The items are rated as disagree (Score 0), partially agree (Score 1) or strongly agree (Score 2). A comment section was provided in the questionnaire for participants to provide specific suggestions. The participants' feedback and suggestions, if any, were taken into consideration for refining the scripted content before converting it into a multimedia.

### 2.6 Phase 3: Development and validation of educational multimedia

In the third phase, the educational multimedia was developed by an animation artist based on the validated script. The educational video was developed in a two‐dimensional animated form using Adobe Illustrator, Adobe After Effects and Animaker software. The initial draft of the multimedia underwent independent review by the research team (all authors). Based on the consolidated feedback provided by the research team, modifications were communicated by Author 1 to the animation artist. The revised animated video was broadcasted to the research team during a joint meeting to establish consensus among the team members. Subsequently, the video was presented to the participants (seven SLPs and 15 representatives of the general public) for content validation of its understandability and actionability. The animated video was individually shown to each participant in a distraction‐free room through a 14ʹʹ laptop screen, with audio presented over headphones. Validation was conducted using the Patient Education Materials Assessment Tool for Audiovisual Materials (PEMAT‐A/V) questionnaire.^39^ There are 17 items in the PEMAT‐A/V, 13 of which are related to actionability and four to understandability. Each response is given a score of one (for agree), zero (for disagree), or not applicable (no score and noted as not applicable). PEMAT‐A/V has been reported to have strong levels of internal consistency, reliability and construct validity.^39^ Additionally, a comment section was provided at the end of the questionnaire for participants to provide specific suggestions.

### 2.7 Data analysis

The data of the ECVIH instrument was analyzed to obtain content validity index (CVI). Items rated ‘disagree’ were assigned a score of zero. Items rated ‘strongly agree’ and ‘partially agree’ were given a score of one. Both item‐level CVI (I‐CVI) and scale‐Level CVI (S‐CVI) were calculated. I‐CVI is the proportion of experts rating the items as ‘partially agree’ or ‘strongly agree’ ([total no. of partially agreed/strongly agreed items]/[number of experts]). The average of I‐CVI score for each item on the scale is the S‐CVI ([sum of I‐CVI scores in the scale]/[number of items in the scale]). Based on the recommendation by Yusoff,^40^ a CVI value of 0.83 or higher for experts and 0.78 or higher for representatives of the general public was considered satisfactory for the validation process, depending on the sample size of each participant group.

The responses to the PEMAT‐A/V were examined to obtain an understandability and actionability score for the developed multimedia educational video. The understandability score was calculated by dividing the total number of items rated as agreed on understandability items by the number of understandability items rated (determined by excluding the ‘not applicable’ items). The actionability score was calculated in the same manner by including actionability items. For each subscale, the scores were multiplied by 100 to obtain the percentage value. Scores under 70% showed that the information's understandability or actionability was poor, with higher percentages suggesting stronger understandability and actionability.^39^

## 3 RESULTS

The results of the mNGT used to identify themes for the educational multimedia are provided in Table 2. The table presents a list of themes identified at each stage of mNGT. In the initial two steps, 24 themes were generated based on the National Institute of Aging,^33^ along with silent generation of ideas by the research team. These 24 themes were narrowed to nine following the clarification stage. The order of presentation of these themes, as determined by the ranking process, is depicted in the last column of Table 2.

**Table 2:** List of potential themes identified during modified nominal group technique.

Themes identified from NIA module (Step 1), silent generation of ideas (Step 2) and round robin (Step 3)	Themes identified after clarification (Step 4)	Presentation order of the themes after ranking (Step 5)
About cognitive health Domains of cognition Importance of cognition for everyday life Consequences of having cognitive disorder Risk factors for dementia Healthy cognitive ageing Causes of dementia Signs and symptoms of dementia Different stages of dementia Mild cognitive impairment versus dementia Preventing dementia/Alzheimer's disease Diet and dementia Physical activity to prevent dementia/Alzheimer's Cardiometabolic factors and dementia/Alzheimer's Cognitive training to prevent dementia Yoga and cognitive health Cognitive stimulation Cognitive reserves Mindfulness and cognition Metacognition for healthy cognition Ways to enhance metacognition Stroke: signs, causes, treatment Forgetfulness: normal or not Aging brain and thinking	Introduction to cognitive health Normal cognitive ageing Risk factors for pathological cognitive aging Pathological cognitive ageing Importance of cognitive health Importance of metacognition Approaches to enhance cognitive reserves Methods to improve metacognition Cognitive reserves	1. Introduction to cognitive health 2. Importance of cognitive health 3. Normal cognitive aging 4. Pathological cognitive aging (MCI and dementia) 5. Risk factors for pathological cognitive aging 6. Cognitive reserve 7. Approaches to enhance cognitive reserves 8. Importance of metacognition 9. Methods to improve metacognition

Abbreviations: NIA, national institute of aging; MCI, mild cognitive impairment.

Based on these nine themes, the first version of the script was drafted and reviewed by the research team. The feedback received during the first round of review from the research team (Authors 3–6) included suggestions for using shorter phrases for simplicity, incorporating examples when discussing the importance of cognition and signs of pathological cognitive aging, mentioning cognitive skills that remain unchanged or increase with age alongside skills that decline with normal cognitive aging, and removing excessive information about the rationale behind each risk factor. Furthermore, the research team proposed writing the script in a dyadic manner to reflect a two‐way interaction between the audience and narrator. The revised script then received consensus from the research team during the second group discussion. The researchers found the revised script to be balanced with respect to the information and ready to effectively communicate essential knowledge about cognitive aging to the intended audience. The key elements of the finalized script, along with the theme‐specific details, strategies used for conveying the content and duration of each segment, are described in Table 3.

**Table 3:** Description of the educational multimedia.

Sections	Themes	Key issues addressed	Strategy used to deliver content	Duration (min)
1	Introduction to cognitive health	Explanation on what is cognition and cognitive health. Explanation of different cognitive domains with examples from everyday life.	Descriptive with examples	1.15
2	Importance of cognitive health	Role of cognition in everyday activities, complex activities, educational activities and occupational activities with examples.	Descriptive with examples	1.10
3	Normal cognitive aging	Age‐related changes in each cognitive function with examples from everyday life.	Analogy based with examples	2
4	Pathological cognitive aging	Mild cognitive impairment—signs and symptoms. Dementia—signs and symptoms.	Analogy based with examples	2.15
5	Risk factors for pathological aging	Description of the risk factors like genetic, family and medical history, brain injury, alcohol or drug consumption, smoking habits, poor physical exercise, poor diet, socialization, education.	Descriptive with examples	3
6	Cognitive reserve	Role of cognitive reserves in cognitive aging.	Descriptive	1
7	Approaches to enhance cognitive reserves	Description of approaches to enhance cognitive reserves like exercising regularly, eating healthy, managing stress, good sleeping habits, managing cardiovascular health, engaging in brain fitness.	Analogy based with examples	3
8	Importance of metacognition	Importance of being aware of cognitive health.	Descriptive with examples	1
9	Methods to improve metacognition	Importance of cognitive assessment, self‐monitoring, family and friends feedback and available cognitive training programs.	Descriptive with examples	1

The results pertaining to the content validation of the educational script done using the ECVIH questionnaire^35^ are depicted in Table 4. The table displays agreement ratings of both experts and representatives of the general public, along with the I‐CVI and S‐CVI scores for the educational script. The content of the script attained minimum acceptable agreements among the experts (CVI: 0.93) and representatives of the general public (CVI: 0.86).^40^ No descriptive comments were obtained from the participants in the comment section of the questionnaire.

**Table 4:** The relevance ratings on the item scale by participants.

Items from the instrument	Experts (SLP) in agreement (*n* = 7)	Expert I‐CVI	Representative of general public in agreement			Total general public in agreement	General public I‐CVI
			YA (*n* = 5)	MAA (*n* = 5)	OA (*n* = 5)		
Objective (Purposes, goals, targets)							
1. ‘Contemplates the proposed theme’	7	1	5	4	5	14	0.93
2. ‘Suits the teaching‐learning process’	7	1	4	4	5	13	0.87
3. ‘Clarifies doubts on the addressed theme’	7	1	4	4	4	12	0.80
4. ‘Provides reflection on the theme’	6	0.85	4	4	4	12	0.80
5. ‘Encourages behaviour change’	6	0.85	4	4	5	13	0.87
S‐CVI/Ave		0.94	S‐CVI/Ave				0.85
Structure/presentation (Organization, structure, strategy, consistency, and sufficiency							
6. ‘Language appropriate to the target audience’	6	0.85	5	5	4	14	0.93
7. ‘Language appropriate to the educational material’	7	1	4	5	5	14	0.93
8. ‘Interactive language, enabling active involvement in the educational process’	6	0.85	4	4	4	12	0.80
9. ‘Correct information’	7	1	4	4	5	13	0.87
10. ‘Objective information’	6	0.85	5	3	4	12	0.80
11. ‘Enlightening information’	6	0.85	4	4	5	13	0.87
12. ‘Necessary information’	7	1	5	4	3	12	0.80
13. ‘Logical sequence of ideas’	7	1	4	4	4	12	0.80
14. ‘Current theme’	7	1	5	4	4	13	0.87
15. ‘Appropriate text size’	7	1	5	4	3	12	0.80
S‐CVI/Ave		0.94	S‐CVI/Ave				0.85
Relevance (Significance, impact, motivation and interest)							
16. ‘Encourages learning’	6	0.85	4	5	5	14	0.91
17. ‘Contributes to knowledge in the area’	7	1	5	5	4	14	0.95
18. ‘Arouses interest in the theme’	6	0.85	4	3	5	12	0.82
S‐CVI/Ave		0.9	S‐CVI/Ave				0.89
Total S‐CVI/Ave		0.93	Total S‐CVI/Ave				0.86

Abbreviations: I‐CVI, item‐level content validity index; MAA, middle‐aged adults; OA, old adults; S‐CVI, scale‐level content validity index; SLP, speech language pathologist; YA, young adults.

The validated script was then used to develop the animated video, which was first independently reviewed by the research team (all authors). The suggestions of the research team included aspects like alternating between multiple angles of the main character (front view and lateral view) and the audience (front view and lateral view), adding more movements to the main character, such as hand gestures, making the character walk around the stage, using warm colours throughout the scenes, removing any distracting and unnecessary gestures or animations in the video, reducing the rate of speech of the voice‐over, adding more pauses across sentences, and providing introduction titles for each segment of the video. The modified version of the video was broadcasted to the research team (all authors) during a joint session wherein no further suggestions were obtained, and consensus was reached among the members regarding the video's attributes. The rate of speech, along with well‐timed pauses and clear segment titles, was considered appropriate. Overall, the group found the revised video to be engaging and lively, with optimal comprehension and smooth flow of information, making it easier for the audience to connect with the content.

The multimedia was then presented to the participants for evaluating its understandability and actionability using PEMAT‐A/V.^39^ The descriptive statistics of the PEMAT‐A/V individual item ratings are provided in Table 5. In the understandability section, the majority of the experts and representatives of the general public reported that the content of the educational video had a clear and evident purpose (Item 1), with appropriate word choice and style (Items 3, 4, 5), organization (Items 8, 9, 10, 11), and appropriate use of photographs (Item18). Most participants indicated that Item 19 of the PEMAT‐A/V, which deals with tabular depictions in multimedia, was not applicable in the present context because there were no tabular representations in this video. Overall, the developed video received an understandability score of 92.8% from experts and 98.8% from representatives of the general public. In the actionability section, the majority of participants rated that the video clearly defined an action that a user could take (Item 20), directly addressed the user (Item 21), and appropriately depicted actions into manageable steps (Item 22). Item 25, which deals with tabular depictions in multimedia, was rated as not applicable by most participants (100%), as the video contained no visual representation of information in the form of table. Overall, the developed video received an actionability score of 100% from both, experts and representatives of general public. In the comments section, the educational multimedia received positive feedback from the participants. Few of the excerpts from participants are mentioned below:

Impeccable educational video, clearly has high production value. Astounding description of cognitive health. (YA4)

It was interesting. I would like to know more about my cognitive status. (MAA2)

Really educative should be followed by all persons specially seniors to keep their brain in active and healthy condition. (OA5)

Really appreciate the concept and creativity of the video. Such awareness‐oriented content would definitely benefit the public. A typical educational video with a monologue would seem monotonous & uninteresting to the viewer. However, this video stands out due to its engaging graphical representations capturing viewer's attention. I personally liked the way concepts of healthy and pathological aging were simplified through portrayal of aging brain, slipping brain, and armoured brain without invoking negative fear or anxiety among the viewers. The video would definitely encourage viewers to ponder and reflect where they lie within in the aging spectrum. (SLP1)

**Table 5:** Descriptive statistics of the PEMAT‐A/V.

PEMAT‐A/V factors and items	Frequency of experts in %			Frequency of representatives of general public in %		
	Disagree	Agree	N/A	Disagree	Agree	N/A
*Subscale: Understandability*						
Topic: Content						
1. ‘The material makes its purpose completely evident’	0	100	0	0	100	0
Topic: Word choice and style						
3. ‘The material uses common, everyday language’	14.2	85.7	0	0	100	0
4. ‘Medical terms are used only to familiarize audience with the terms. When used, medical terms are defined’	0	100	0	0	100	0
5. ‘The material uses the active voice’	0	100	0	0	100	0
Topic: Organization						
8. ‘The material breaks or “chunks” information into short sections’	0	100	0	6.7	93.3	0
9. ‘The material's sections have informative headers’	14.2	85.7	0	0	100	0
10. ‘The material presents information in a logical sequence’	0	100	0	0	100	0
11. ‘The material provides a summary’	14.2	85.7	0	0	100	0
Topic: Layout and design						
12. ‘The material uses visual cues (e.g., arrows, boxes, bullets, bold, larger font, highlighting) to draw attention to key points’	28.5	71.4	0	6.7	93.3	0
13. ‘Text on the screen is easy to read’	0	100	0	0	100	0
14. ‘The material allows the user to hear the words clearly (e.g., not too fast, not garbled)’	0	100	0	0	100	0
Topic: Use of visual aids						
18. ‘The material uses illustrations and photographs that are clear and uncluttered’	14.2	85.7	0	0	100	0
19. ‘The material uses simple tables with short and clear row and column headings’	0	0	100	0	0	100
Understandability score (%)	92.85%			98.8%		
*Subscale: Actionability*						
20. ‘The material clearly identifies at least one action the user can take’	0	100	0	0	100	0
21. ‘The material addresses the user directly when describing actions’	0	100	0	0	100	0
22. ‘The material breaks down any action into manageable, explicit steps’	0	100	0	0	100	0
25. ‘The material explains how to use the charts, graphs, tables or diagrams to take actions’	0	100	0	0	0	100
Actionability score (%)	100%			100%		

Abbreviation: PEMAT‐A/V, Patient Education Materials Assessment Tool for Audiovisual Materials.

**Figure 2:**
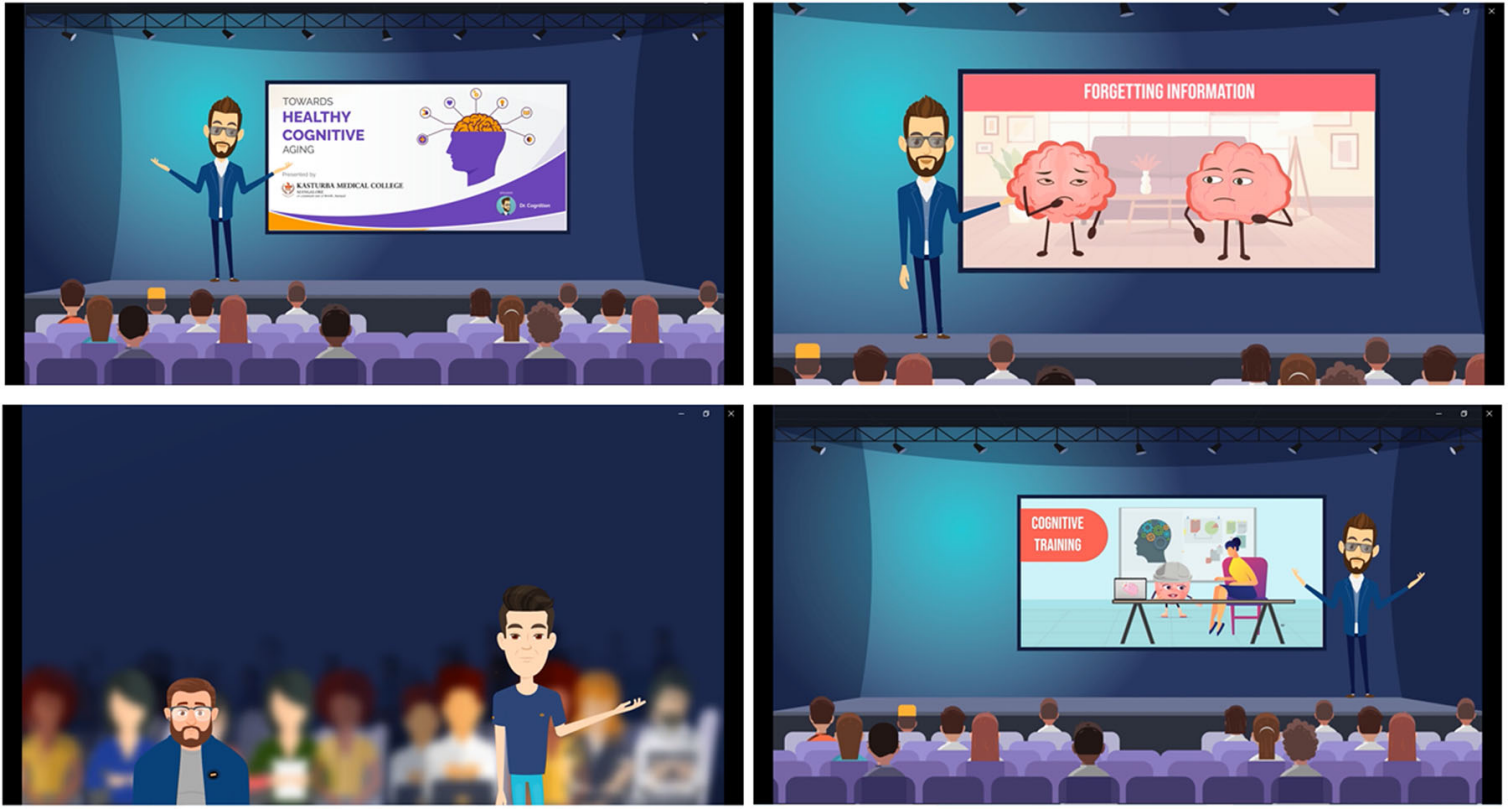
Snippets of the educational multimedia on cognitive health.

The snippets of the finalized version of the educational multimedia, which had a total duration of 15.38 min, are provided in Figure 2.

## 4 DISCUSSION

To address the concerning global and national forecasts of cognitive disorders such as Alzheimer's and dementia,^12^, ^13^ it is crucial to improve cognitive HL through the use of scientifically sound educational resources. In a similar direction, the present study focused on scientifically scripting and validating content for public HL in cognitive health, which was then transformed into an educational multimedia format. The following section describes the fundamental elements of educational multimedia, such as its content and delivery method, by drawing upon relevant literature and its underlying theoretical principles. Additionally, reflections and recommendations for future projects in this area are provided.

### 4.1 Content‐based aspects of educational multimedia

The content of the developed educational script was validated using ECVIH,^35^ and demonstrated optimal validation scores by experts and representatives of the general public across its different domains, such as objective, structure, presentation and relevance. These findings are congruent with the results of other methodologically similar studies that have demonstrated acceptable validation scores for educational multimedia in other health domains.^29^, ^36^ A satisfactory agreement on the ‘objective’ domain implies that the developed educational material appropriately represents the information about cognitive health, supports the teaching–learning process, clears up misconceptions about cognitive health, promotes reflection on cognitive health and inspires behaviour change for healthy cognition, as opined by the experts and representatives of general public for the educational multimedia. These satisfactory results could be attributed to the theoretical foundations considered when framing the script of the present educational multimedia. The structure and content of the script were based on the integrated HL framework.^1^ According to the framework, the four dimensions that any HL could include are the ability to access, comprehend, interpret and enable informed decisions related to health information, risk factors and determinants of health. These aspects are important for healthcare, disease prevention and health promotion. Since the HL domain of the current study intended to advocate the prevention of cognitive disorders by promoting the importance of cognitive health, it was important to ensure that the participants understand the importance of cognitive health, appraise the risk factors which pose threat to cognitive health, evaluate and reflect on one's cognitive health status in the background of the norms, and be aware of appropriate measures to prevent any cognitive disorders later in life. Accordingly, the developed educational material in the present research was centred around nine themes addressing these areas, which were informed by the cognitive health module provided by the National Institute of Aging^33^ and the systematic method, such as mNGT. The thematic aspects of the script were reinforced by the modules given by the National Institute of Aging^33^ about public information on cognitive health such as, ‘cognitive health and older individuals’, ‘how the aging brain affects thinking; forgetfulness: normal or not?’, ‘memory, forgetfulness and aging: what's normal and what's not?’, ‘tips to boost your health as you age’, ‘making healthy lifestyle choices may reduce your risk of dementia’, ‘preventing Alzheimer's disease: what do we know?’, and ‘what do we know about healthy aging?’. Leveraging the resources provided by recognized organizations that offer comprehensive information created and verified by experts in the field may have proven helpful in assisting with the selection of themes and scripting the draft. In addition, using a structured consensus method, such as mNGT,^32^ which aims to foster agreement among participants during idea generation by ensuring balanced participation from group members,^41^ may have played a role in identifying pertinent themes. This method has been successfully used in previous research to inform key areas of educational programmes.^42^, ^43^, ^44^, ^45^

With regard to the ‘structure and presentation’ and ‘relevance’, the educational material was considered to be organized, structured, strategic, consistent, sufficient, encourages learning, contributes to knowledge about cognitive health, and sparks interest in it. The satisfactory agreement on the structure and presentation of the educational material by experts and representatives of the general public could be attributed to the ‘TED‐like’ strategy used to communicate the present HL content. TED and TED‐like talks are important mediums for disseminating scientific knowledge to the general audience.^46^ Educational interventions in a ‘TED‐like’ style have proven effective in other healthcare domains.^34^, ^47^ The educational content of the current educational multimedia was scripted in a ‘TED‐like’ style that included a lead speaker named ‘Dr. Cognition’ delivering a talk titled ‘Towards healthy cognitive aging’ using a PowerPoint presentation format. Further, informational value, relevance, and social connections are the primary determinants of user acceptance of any HL‐related video.^48^ The educational script in the present study was written in a format that reflects dyadic communication between a speaker and his audience to enhance social connections. Additionally, each section of the script included appropriate examples from everyday life to augment comprehension of the intended message and enhance information value. It seems that the inclusion of such strategies might have contributed to optimal CVI score for the domain of ‘structure and presentation’ and ‘relevance’ of the present HL material.

### 4.2 Delivery‐based aspects of the educational multimedia

The present content was chosen to be delivered through a multimedia format. The use of multimedia in health education is generally justified by the notion that creating a mental representation through multisensory experiences increases the likelihood that information will be remembered, and as a result, the likelihood that recommendations will be followed.^49^ Multimedia educational materials have been reported to be more interesting to participants than printed materials.^48^ To validate the robustness of the present multimedia, we used PEMAT‐A/V to assess its understandability and actionability.^39^ Overall, the developed multimedia education received high understandability and actionability scores from both experts (92.8% and 100%, respectively) and representatives of the general public (98.8% and 100%, respectively). The understandability ratings showed that the module was perceived to be intelligible and included concise information that was simple to grasp. The module employed everyday English rather than technical terms, and ensured that any medical terminology was accompanied by subtitles and definitions to avoid any misinterpretation of the content. Though the module did not use any tabular content, it made use of appropriate visual aids, such as photographs and illustrations, to aid easier understanding among the audience. The actionability score implies that the module effectively communicates with its audience and outlines concrete actions they could adopt for their cognitive well‐being.

### 4.3 Reflections and recommendations for future versions

Although all the parameters of ECVIH met the required CVI scores, the authors further examined the data to identify elements within the ECVIH that received relatively lower agreement scores than others. Items such as ‘provides reflection on the theme’ (Item 4), ‘encourages behaviour change’ (Item 5), ‘interactive language, enabling active involvement in the educational process’ (Item 8), ‘objective information’ (Item10), ‘enlightening information’ (Item 11) and ‘arouses interest in the theme’ (Item18) seem to have received CVI scores less than 0.9 by both the participant groups. To strengthen these specific areas, additional versions of the developed multimedia could include testimonies from people with cognitive impairments or their caregivers. Including celebrity testimonials has been shown to be effective in engaging participants in dementia awareness campaigns.^50^ Further, inclusion of research data that exhibit the benefits of cognitive engagement on cognitive reserves or postponement of dementia, could aid in enhancing the objectivity of the content. The developed educational multimedia used TED‐like delivery style, portraying interactions between the main character of the video, Dr. Cognition, and his audience. Additionally, future work could explore the integration of such multimedia with AI chatbots, allowing viewers to pause the video and interact with the character to clarify their specific queries.

The present research has certain limitations. The inclusion of representatives of the general public, especially those with some association with individuals with cognitive disorders, could have provided a more profound understanding of certain aspects related to cognitive health. Additionally, the current validation process exclusively involved public representatives from a specific societal segment, specifically those of middle socioeconomic status. Including representatives from all segments of society could enhance the overall applicability of the current educational multimedia. Nevertheless, this robust and scientifically sound educational multimedia could be a beneficial contribution to the public health interventions related to cognitive health. The studies evaluating efficacy of this educational multimedia across various demographics like age, clinical conditions and educational backgrounds are in progress. Further, the validated educational script can serve as standardized educational material that can be adopted and translated to regional languages by researchers and clinicians to reach a larger population.

## 5 CONCLUSION

The present study focused on developing and validating educational multimedia on cognitive health based on integrated health frameworks, educational modules on cognitive health by the National Institute of Aging, and an mNGT approach. The script of the educational multimedia received optimal satisfactory agreements from experts (CVI: 0.93) and representatives of the general public (CVI: 0.86) on all items and sections of ECVIH. In its multimedia format, the educational content received high understandability (experts: 92.8%, representatives of general public: 98.8%) and actionability (experts: 100%, representatives of general public: 100%) scores using the PEMAT‐A/V. Overall, the educational multimedia was well received by the experts and representatives of the general public. The successful validation results suggest the significance of employing this educational multimedia as a potent instrument to foster awareness and comprehension of cognitive health among diverse audiences. Besides evaluating the efficacy of the present multimedia and reproducing it in different languages, future versions of the developed multimedia could include testimonies from people with cognitive impairments or their caregivers, and include research data on the benefits of cognitive engagement on cognitive reserves.

## AUTHOR CONTRIBUTIONS

Aysha Rooha, Shreya Shetty, Gagan Bajaj and Nidhi Lalu Jacob conceptualized and designed the study. Aysha Rooha and Shreya Shetty collected the study data. All the authors were involved in the analysis and interpretation of the data. Aysha Rooha, Shreya Shetty and Gagan Bajaj drafted the manuscript. Nidhi Lalu Jacob, Vinitha Mary George and Jayashree S. Bhat edited the manuscript. All the authors read and approved the final manuscript.

## CONFLICT OF INTEREST STATEMENT

The authors declare no conflict of interest.

## ETHICS STATEMENT

Ethical approval was obtained from the Institution's Ethics Committee (IEC KMC MLR—05‐2022/171). Informed consent was obtained from all participants before the study.

## Data Availability

The data that support the findings of this study are available from the corresponding author upon reasonable request.
